# Supplementary material for: Development and Field Validation of Lidocaine-Loaded Castration Bands for Bovine Pain Mitigation
Source: Animals (Basel). 2020 Dec 10;10(12):2363. doi: 10.3390/ani10122363 (PMC7763166; doi:10.3390/ani10122363)
Supplement: Supplementary file 1 [file animals-10-02363-s001.pdf]

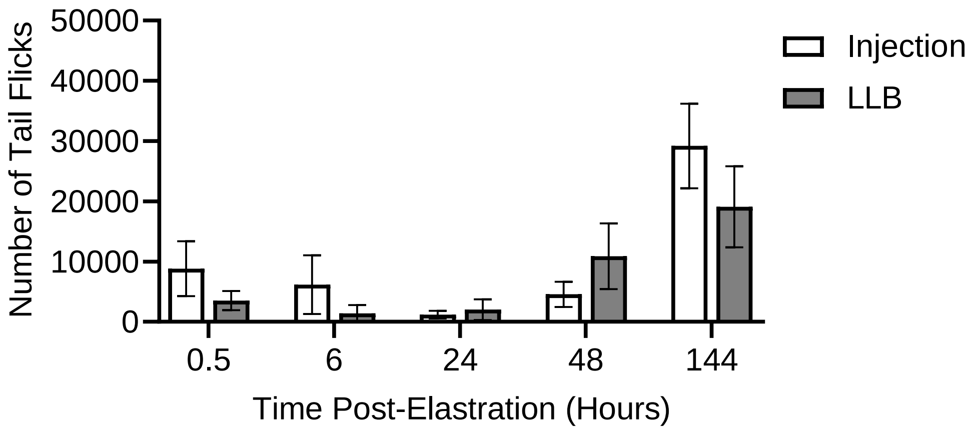

Supplemental Figure S1. Number of tail flicks for control (lidocaine injection) or treated (LLB) animals over time. Time indicates the duration the band was on the animal. Tail flicks were measured using a pedometer. Data reflect small green bands only. Bars represent the mean ± standard error of the mean for three independent replicates per treatment per time point.

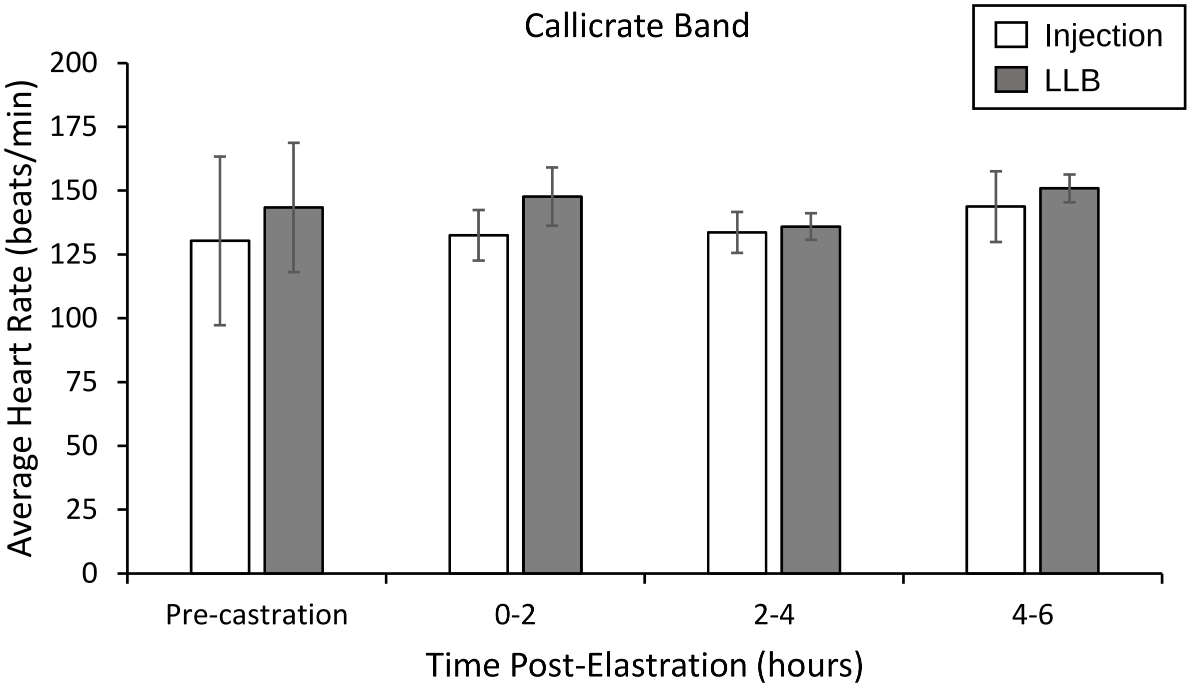

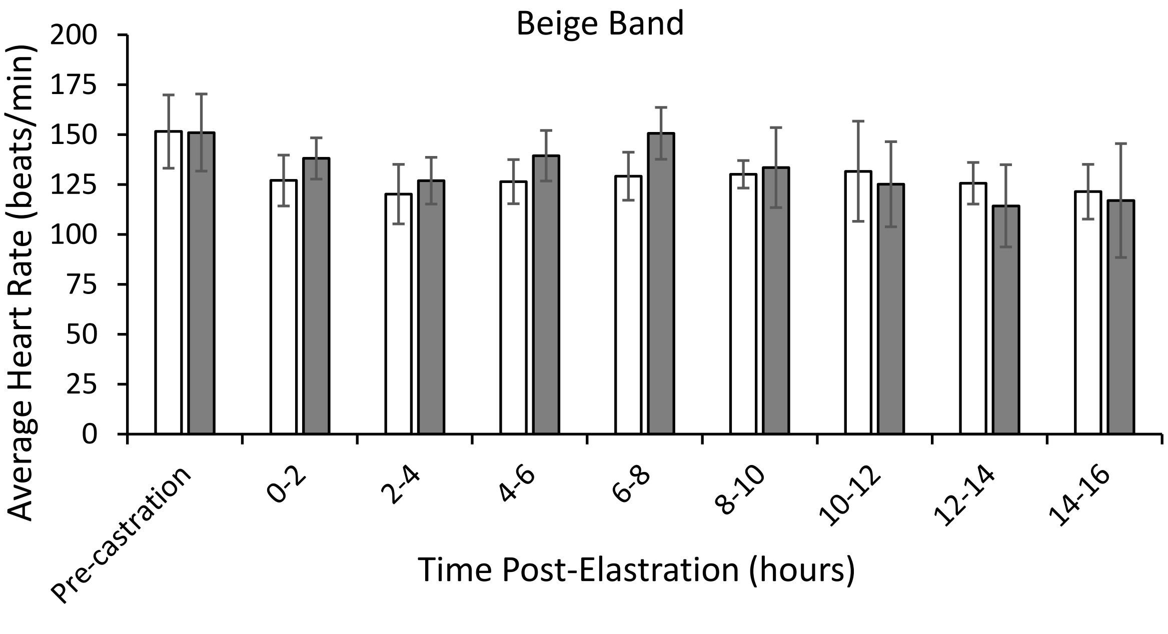

Supplemental Figure S2. Average heart rate for control (lidocaine injection) or treated (LLB) animals over time. Time indicates the duration the band was on the animal. Data reflect large Callicrate bands and medium beige bands. Bars represent the mean ± standard error of the mean for three independent replicates per time point.

Supplemental Methods.

To measure tail flicks, a Meilen model Mj001 pedometer (Mobil Shenzhen Electronics Co., Ltd.) was attached to the tails of the animals in trial 1 (the Green-banded animals) at t = 0 using vet wrap. Data was downloaded at each time point (0.5, 6, 24, 48, 144 hours post-elastration).

To measure average heart rate of the beige-banded animals (trial 2) and the Callicrate-banded animals (trial 3), a SmartSync heart rate data logger (Model WM100, Oregon Scientific, USA) was affixed to the animals at t=0 using the manufacturer-supplied harness and reinforced with duct tape. Data was downloaded for the following time intervals: 2 hours pre-elastration and 0-2, 2-4, 4-6, 6-8, 8-10, 10-12, 12-14, and 14-16 hours post-elastration.
